# Second victims among emergency medical dispatchers in Germany: a cross-sectional study (SeViD-VII)

**DOI:** 10.1186/s12245-025-01084-y

**Published:** 2025-12-22

**Authors:** Victoria Klemm, Reinhard Strametz, Thomas Neusius, Matthias Raspe, Rafael Trautmann, Marc Gistrichovsky, Rainer Petzina, Stefan Bushuven, Hartwig Marung

**Affiliations:** 1 Department of Internal Medicine, Speciality Network: Infectious Diseases and Respiratory Medicine, Charité – Universitätsmedizin Berlin, Corporate Member of Freie Universität Berlin, Humboldt-Universität zu Berlin, and Berlin Institute of Health, Charitéplatz 1, 10117 Berlin, Germany; 2 Wiesbaden Institute for Healthcare Economics and Patient Safety (WiHelP), RheinMain University of Applied Sciences, Bleichstraße 44, 65183 Wiesbaden, Germany; 3 German Society of Paramedic Science (DGRe e.V.), Weststraße 6, 52074 Aachen, Germany; 4 Control Centers Association (FVLST e.V.), Blomberger Weg 60, 32657 Lemgo, Germany; 5 Department Health Sciences, MSH Medical School Hamburg, Am Kaiserkai 1, 20457 Hamburg, Germany; 6 Training Center for Emergency Medicine (NOTIS e.V.), Vilstalstraße 26A, 87459 Pfronten, Germany; 7 Department of Anesthesiology and Critical Care, Medical Center - University of Freiburg, 79106 Freiburg, Germany

**Keywords:** Second victim, Emergency medical dispatchers, Emotional distress, Psychological distress, Patient safety, Occupational health

## Abstract

**Background:**

Emergency Medical Dispatchers (EMDs) play a critical role in coordinating emergency responses while being remotely exposed to distressing incidents. The Second Victim Phenomenon (SVP) refers to emotional distress experienced by healthcare professionals following adverse events; however, its prevalence and impact among EMDs remain poorly understood. This study examines the prevalence, symptom burden, and support preferences related to SVP among EMDs in Germany.

**Methods:**

A cross-sectional survey was conducted using the validated SeViD-questionnaire, supplemented with demographic items. The survey assessed SVP prevalence, symptom severity, and preferred support measures. A web-based survey was distributed via professional networks. Descriptive statistics summarized sample characteristics and binary logistic regression was used to identify predictors of SVP and symptom burden.

**Results:**

Among 407 respondents, 315 completed the questionnaire (completion rate: 78%). More than half (62.2%) of participating EMDs identified as Second Victims, despite limited prior awareness of the phenomenon. Traumatic events such as patient deaths or suicides and aggressive behavior were central triggers. SVP was linked to symptoms including emotional strain and difficulties concentrating, with neuroticism associated with greater symptom load (*p* = 0.02). Binary logistic regression suggested that higher scores of the personality trait openness and professional experience increase SVP risk (*p* < 0.01). Although many affected EMDs refrained from seeking help, those who did often relied on their friends and family for support. Immediate time off, structured debriefing, and professional counseling emerged as the most valued support strategies.

**Conclusions:**

SVP is highly prevalent among EMDs, highlighting the need for targeted support strategies. Raising awareness, integrating SVP education into dispatcher training, and establishing accessible psychological support programs are essential to improve well-being and resilience in this professional group.

**Supplementary Information:**

The online version contains supplementary material available at 10.1186/s12245-025-01084-y.

## Introduction

Emergency medical dispatchers (EMDs) play a key role in coordinating emergency responses, serving as the link between individuals in crisis and on-site emergency teams [1]. Unlike emergency medical technicians, who are physically present at the scene, EMDs operate remotely, often experiencing helplessness as they oversee unfolding emergencies [2]. Despite this distance, they are exposed to traumatic events and distressing calls from witnesses in shock or distress [3–5]. Limited information, resulting from the absence of visual cues and the frequent incompleteness or inaccuracy of caller reports, as well as time pressure adds substantially to their emotional burden [6, 7]. Under these conditions, dispatchers must make high-pressure decisions, provide reassurance and guidance to distressed callers, and coordinate responding teams. These stressors contribute to EMDs developing the Second Victim Phenomenon (SVP). A Second Victim is “any health care worker, directly or indirectly involved in an unanticipated adverse patient event, unintentional healthcare error, or patient injury and who becomes victimized in the sense that they are also negatively impacted” [8]. The SVP has been the subject of numerous studies. Systematic reviews have reported that the prevalence of SVP varies between 10% and 58% across the analyzed studies [9, 10]. Second Victims may experience a spectrum of consequences, primarily manifesting as psychological reactions such as anxiety and a diminished sense of self-confidence [11], as well as physical symptoms including but not limited to back pain and headaches [12–14]. Detrimental reactions within organizations are also prevalent, often evidenced by health professionals` increased absenteeism and intentions to leave their positions [15]. Moreover, SVP compromises patient safety and the quality of care. Instances of healthcare providers engaging in defensive medical practices [16, 17] and elevated error rates resulting from the development of post-traumatic stress disorder (PTSD) subsequent to a severe incident have been documented [11]. While previous SeViD-studies (Second Victims in German-speaking countries) have examined SVP among physicians, nurses, midwives, and paramedics, little is known about its prevalence in EMDs [12–14, 18–21]. This group differs substantially from other first responders, as they are exposed to medical emergencies exclusively via auditory input, must make rapid decisions with limited contextual information, and carry significant responsibility without being physically present at the scene. EMDs work in urban or rural areas, during shifts or regular working hours, and may or may not hold a leadership role within their teams. In the literature, these factors have been associated with increased stress levels among emergency medical services’ staff in general [22–25]. These unique working conditions may shape both the risk of experiencing SVP and the overall symptom load. Given this distinct professional context, a separate investigation of EMDs provides insights into how SVP manifests across different roles in emergency medical services. Furthermore, this research may contribute to the development of targeted interventions to support EMDs affected by SVP.

This study aims to assess the prevalence, symptom load, and preferred support strategies among German EMDs. We hypothesize that the majority of EMDs in Germany have experienced SVP, the most common key events leading to SVP are events that negatively affect patient safety, most EMDs who have experienced SVP have sought support from their own colleagues, the length of service is a predictor of the SVP, and EMDs who have experienced SVP prefer different support measures than those who have not yet experienced it. Additionally, we explore demographic, workplace, and personality factors associated with SVP, as well as the impact of support in mitigating symptoms. This study provides initial insights into SVP among EMDs, informing future interventions.

## Methods

### Study instruments

The SeViD-questionnaire by Strametz et al. is a content-validated instrument designed to assess the prevalence, symptom burden, and coping strategies related to the Second Victim Phenomenon among healthcare professionals [26]. A systematic literature search was conducted to develop the questionnaire. It consists of three domains and 40 items including general experience with the SVP, prevalence of second victim symptoms and second victim support strategies. The domain “general experience with SVP” included seven questions aiming to explore the awareness of the SVP, the prevalence, incidence, key event leading to SVP, support received after experiencing SVP, the most important support groups after the experience, and the self-perceived time of recovery. The second domain “second victim symptoms” consisted of 20 possible symptoms where the participants were asked to rate the severity of their symptoms on a three-point Likert-scale (not pronounced at all, weakly pronounced, strongly pronounced). The last domain “second victim support strategies” aimed to explore the ratings of 13 possible support measures on a four-point Likert-scale (not helpful at all, rather not helpful, rather helpful, very helpful). The SeViD-questionnaire itself does not include demographic questions so that it can be applied across professional groups within the health care setting [26]. Therefore, we added ten items regarding sociodemographic data. Context-specific items included the type of formal medical education, type of emergency medical services’ system (rural vs. urban), disposition areas, and the population size covered. These context-specific items were selected in consultation with the German Society of Paramedic Science to ensure their relevance for the professional context of EMDs. In addition, given that the SARS-CoV-2 pandemic may have increased the likelihood of incidents involving medical errors, we included an item asking whether key incidents were associated with the pandemic. Since the core SeViD-questionnaire was not altered, no additional pilot testing or content validation was performed.

The survey used adaptive questioning so that items from the symptoms’ domain were displayed only to participants indicating that they had experienced specific incidents. The participants were able to move backwards to questions they had answered already and to use a commentary function to give their suggestions at the end of the survey. The SeViD-questionnaire is provided as a Supplementary File 1.

The Big Five Inventory 10 Item Scale (BFI-10) by Rammstedt et al. was used to assess the personality traits of the participants [27]. The Big Five personality traits—openness, conscientiousness, extraversion, agreeableness, and neuroticism—represent five broad dimensions of human personality. Openness reflects imagination and curiosity, conscientiousness relates to self-discipline and organization, extraversion denotes sociability and assertiveness, agreeableness involves empathy and cooperation, and neuroticism describes emotional instability and vulnerability to stress [28]. The BFI-10 is a short version of the Big Five Inventory designed to measure these five major personality traits with two items per dimension [27]. The BFI-10 survey is provided as a Supplementary File 2. The Big Five personality traits have shown to influence susceptibility to emotional stress and were therefore added in this research and previous SeViD-studies to possibly explain occurrence and symptom load of SVP [13, 14, 19–21, 29, 30].

### Data collection and participants of the SeViD-VII survey

**Fig. 1:**
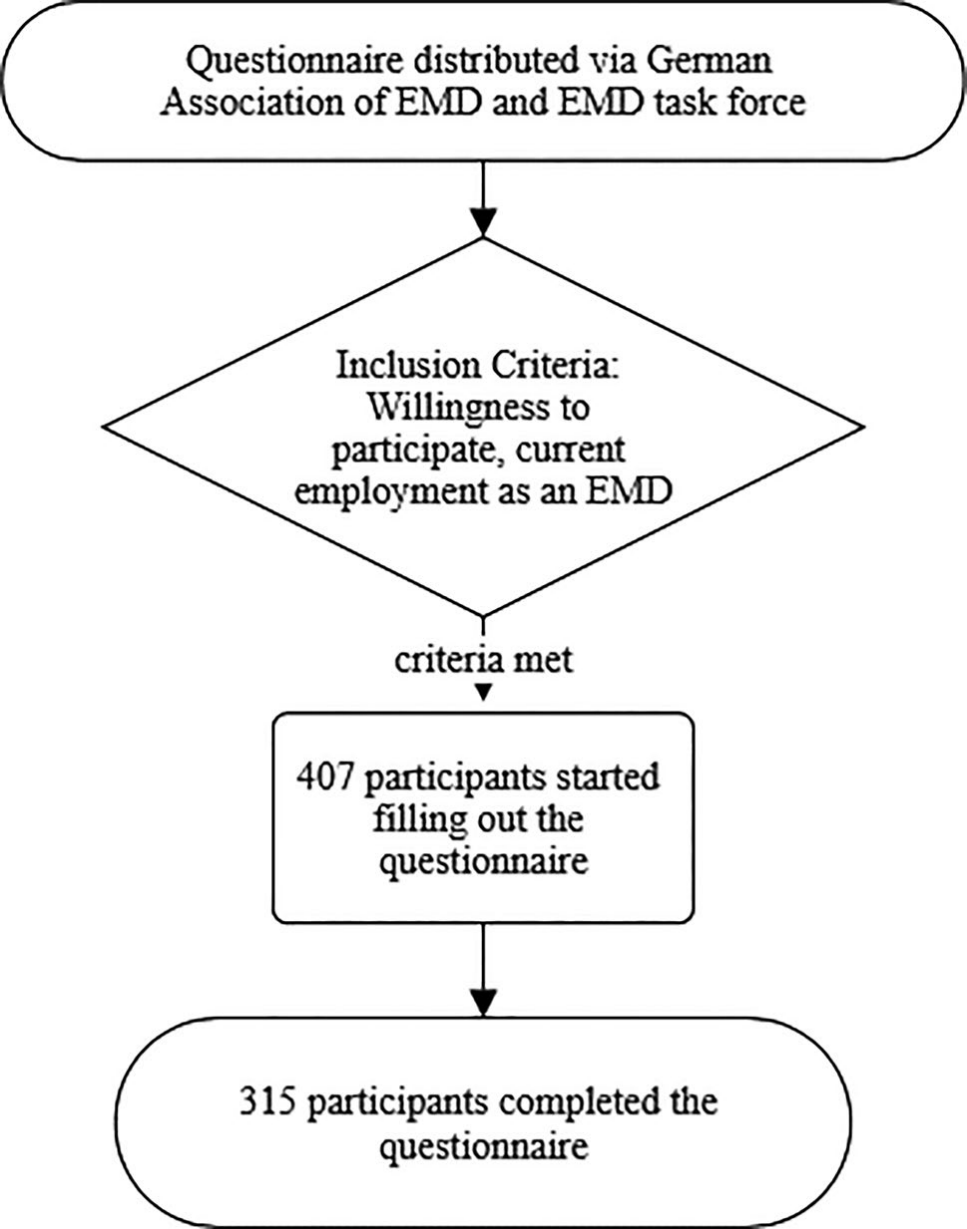
Simplified flowchart of our sampling process

The survey was conducted via web-based distribution of the SeViD questionnaire using the SurveyMonkey platform (San Mateo, CA, USA). Both the German Association of EMD^1^ (“Fachverband Leitstellen”) representing 350 full members and the EMD task force within the German Association for Rescue Sciences^2^ (Deutsche Gesellschaft für Rettungswissenschaften) distributed the link to the survey among their members and via professional networks. Figure 1 shows a simplified flowchart of the sampling process. The invitation letter as well as the following reminders included a brief summary of the purpose of the study and a QR code leading to the web link. Inclusion criteria were willingness to participate and working as an EMD. The study period was from April 27th to August 5th, 2023 and reminders were sent out after two, four and eight weeks. Data collection using convenience sampling was anonymized completely with neither tokens, cookies nor IP addresses stored.

The ethical committee of the medical faculty of the Goethe University Frankfurt granted a waiver for this study, as formal approval was not required (waiver number: 2025-2271-Waiver). The study was conducted in accordance with the Declaration of Helsinki. Informed consent was obtained from all subjects involved in the study in written form.

### Statistical analysis

Descriptive statistics for demographic variables and applied instruments were presented using means (*M*) with standard deviations (*SD*) for interval-scaled variables independent of normal distribution and frequencies (*n*) with percentages (%) for nominal and ordinal scaled variables [31]. To test factors associated with the probability of becoming a Second Victim, we performed a binary logistic regression analysis with the predictors: sex (referent category being female), work experience in years, inhabitants in dispatch-area (reference category being < 100.000 inhabitants), type of dispatch area (reference category being urban), and BFI-10 personality traits. For the dependent variable self-reported Second Victim status, we dichotomized the status in yes/no. Further, we performed a binary logistic regression analysis to test factors associated with symptom load with the predictors: sex (referent category being female), work experience in years, BFI-10 personality traits, support received (no support being the referent category), disposition area (ground-based being the reference category), and type of event (“other” being the reference category). To estimate an overall symptom load, a sum score was calculated. Answers of symptom load were 0 = not pronounced at all, 0.5 = weakly pronounced, 1.0 = strongly pronounced. We defined the answer “I don’t know” as missing value for the symptom load items. To then dichotomize the symptom load with the two categories high vs. low symptom load we performed a median split [32]. After performing this median split, participants with weakly pronounced symptoms were considered part of the group with a high overall symptom load.

In the binary logistic regression models, independent variables were selected based on theoretical considerations and prior findings in the literature. Specifically, we included work experience, gender, type of key event leading to developing SVP, received support, and personality traits, as these factors have been repeatedly associated with susceptibility to SVP and a higher overall symptom load in healthcare professionals identifying as Second Victims [12–14, 18–21]. Additional variables (disposition area, number of inhabitants covered by emergency medicine services) were chosen to capture dispatcher-specific demographic characteristics, aiming to explore their potential influence in this professional context.

We employed list-wise exclusion of cases to handle missing values, ensuring that only complete observations across all variables of interest were included in the analysis.

For the binary logistic regression models we tested the linearity of logit and excluded the BFI-10 personality trait Extraversion since it did not meet the assumption.

We inspected the correlation matrix as well as the variance-inflation factor to test if multicollinearity between the predictors was present. In the correlation matrix, values above 0.7 were considered problematic and a sign of multicollinearity [33]. Variance inflation factor values above “5” were considered problematic, and values above “10” indicated a serious collinearity problem [34, 35]. Results of the multicollinearity diagnostic showed signs of multicollinearity regarding age and work experience. We therefore omitted age from the analysis.

To test any associations between Second Victim status or symptom load and personality dimensions, we used the Big Five Inventory (BFI-10) [27].

Further, we’ve calculated Nagelkerke’s Pseudo-R² and the AUC (area under the curve) to assess the models’ fits and the explanatory power of the chosen predictors as well as their capability to discriminate between the possible binary outcomes [31].

To compare the helpfulness of preferred support measures between participants who encountered SVP at least once in their professional career and participants who did not, we performed the Mann-Whitney-U-Test for independent samples. The measurement of helpfulness of support measures was defined as follows on a five-point Likert-scale: 1 = not helpful at all, 2 = rather not helpful, 3 = rather helpful, 4 = very helpful. Further, we have calculated Pearson’s correlation coefficient (r) to measure the effect size of the significant differences between Second Victims and Non- Second Victims.

Our significance threshold was set at *p* < 0.05 (double-sided). Statistical analysis was performed using SPSS Statistics Version 29 (IBM, New York, NY, USA).

## Results

### Baseline characteristics

Of the 407 participants, 315 completed the questionnaire. Mean time required for completion was 7 min 51 s and the completion rate was 78%. Mean age was 42.1 years (Median (MID) = 42; SD = 9.27; Min = 20; Max = 66). Most participants were male (87.3%, *n* = 275), 12.4% were female (*n* = 39) and one participant did not specify their gender. Work experience ranged between one year and 43 years (Mean = 10.01; MID = 8; SD = 8.35).

Table 1 shows participants` workplace characteristics.

**Table 1:** Workplace characteristics (*n* = 315)

Characteristics	Answer Options	Percentage of Participants (*n*)
Education (multiple choice)	Emergency service training (paramedics, dispatchers)*	98.7 (311)
	Firefighter Training	88.9 (280)
	Higher education (Completed or ongoing education)	4.8 (15)
Disposition area (multiple choice)	Ground-based emergency medical services	91.4 (288)
	Qualified ambulance transport	82.9 (261)
	Disaster management	78.4 (247)
	Fire protection	72.1 (227)
	Heavyweight transport	66.7 (210)
	Intensive care transport	58.1 (183)
	Helicopter-based emergency medical services	52.1 (164)
	Neonatal emergency care	31.7 (100)
	Other	35.2 (111)
Population covered by emergency medical services	< 100,000	1.0 (3)
	100,000-250,000	26.0 (82)
	250,000-500,000	36.5 (115)
	500,000–1,000,000	21.9 (69)
	> 1,000,000	14.6 (46)
Emergency medical services’ setting	Mixed rural and urban	46.3 (146)
	Rural	30.8 (97)
	Urban	22.9 (72)
Work hours	„Nine to five“	5.7 (18)
	Shift work incl. night shifts	75.6 (238)
	Shift work excl. night shifts	1.6 (5)
	Night shift only	0.3 (1)
	Irregular w/o shifts	5.4 (17)
	Other	11.4 (36)

*In Germany, emergency service training encompasses both paramedics and dispatchers. Paramedics typically complete a three-year apprenticeship, with shorter apprenticeships available for assistant roles, or pursue academic studies in rescue sciences. Dispatchers are required to have prior qualification as either a paramedic or firefighter, followed by additional dispatcher-specific training. Consequently, we summarized these groups under the category ‘emergency service training,’ as it is not possible to become a dispatcher without such foundational training

### Second victim status

When asked about their knowledge of SVP, the vast majority had no knowledge of the term (*n* = 258, 81.9%). After explaining the term, 62.2% (*n* = 196) self-identified as Second Victims. Due to this prevalence of SVP among EMDs we accepted the hypothesis that the majority of EMDs have already experienced SVP.

Almost one third of the self-identified Second Victims reported to have experienced the event leading to SVP within the past 12 months at the time of the survey (32.1%, *n* = 101). The most common key event leading to SVP among dispatchers was unexpected death or suicide of a patient (18.4%, *n* = 58), followed by aggressive behavior of patients or relatives (11.1%, *n* = 35). Other key-events were unexpected death/suicide of a colleague (10.5%, *n* = 33), incidents with patient harm (8.3%, *n* = 26), and incidents without patient harm, so-called near misses (7.0%, *n* = 22). Those who marked “other” (7.0%, *n* = 22) specified the key events further in free text as follows, e.g.:

“Flooding catastrophe of 2021” (the flooding in the Ahr Valley region, Germany)

Announced suicide, the caller told me he would kill himself if I hung up the phone

Terrible traffic accident involving the death of an eight-year-old child

“Pediatric resuscitation” (named several times)

**Fig. 2:**
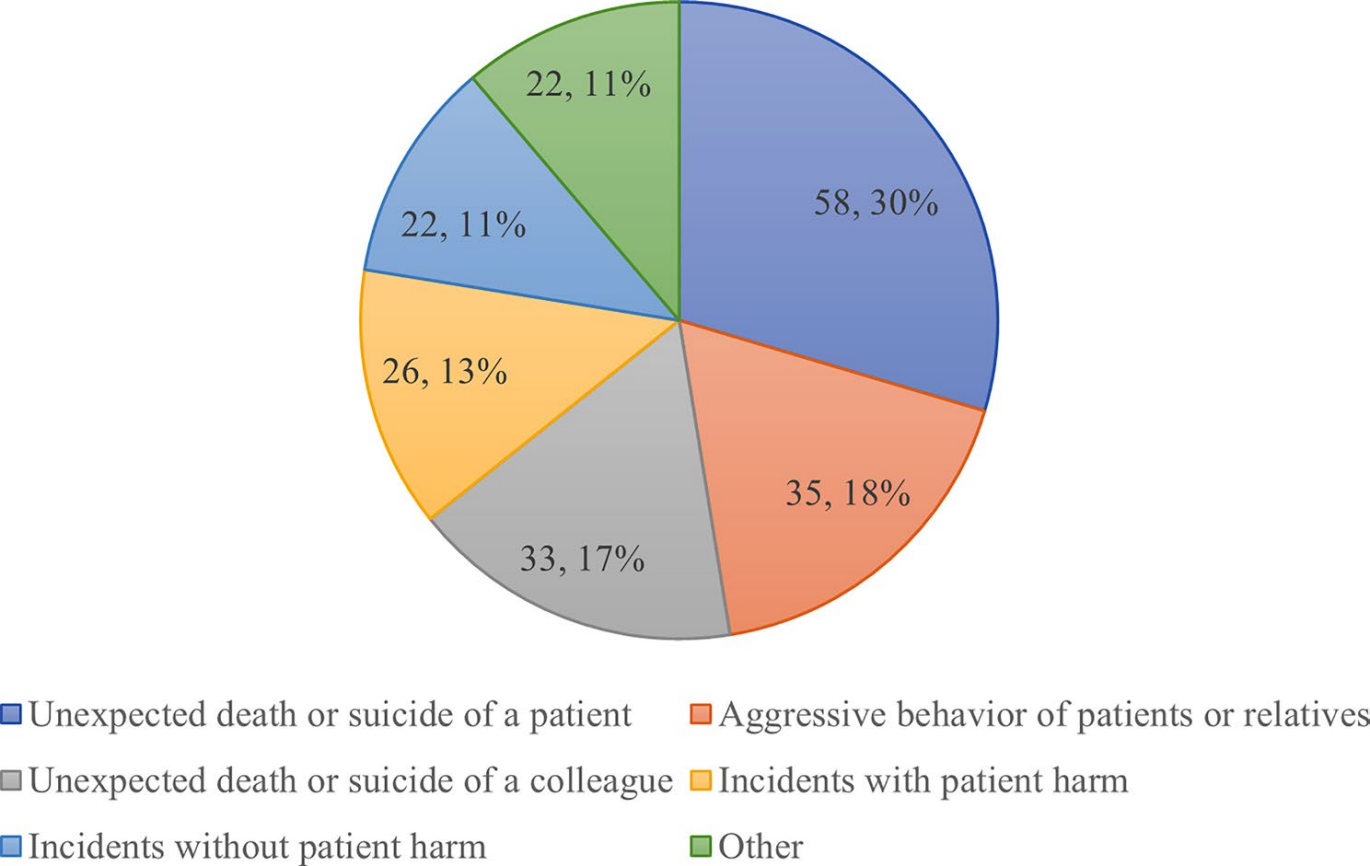
Key events leading to SVP among EMDs

Figure 2 illustrates the distribution of key events leading to SVP in our sample.

Only the most common key event was directly related to patient safety. We had hypothesized that incidents negatively affecting patient safety would primarily lead to SVP among EMDs; however, since neither the second nor the third most frequent key event was associated with patient safety, this hypothesis was rejected.

More than half of the respondents who identified as Second Victims did not seek assistance after the incident (57.7%, *n* = 113), while 39.8% reported receiving support (*n* = 78). Five respondents (1.6%) indicated that they did not receive support despite requesting it. Among those who sought help, family and friends were the main sources of support (42.3%, *n* = 33), followed by professional counseling (30.8%, *n* = 24). We had further hypothesized that most EMDs would turn to their colleagues for support following an SVP incident. Although colleagues were consulted, family and friends played a more prominent role, leading us to reject this hypothesis as well.

**Fig. 3:**
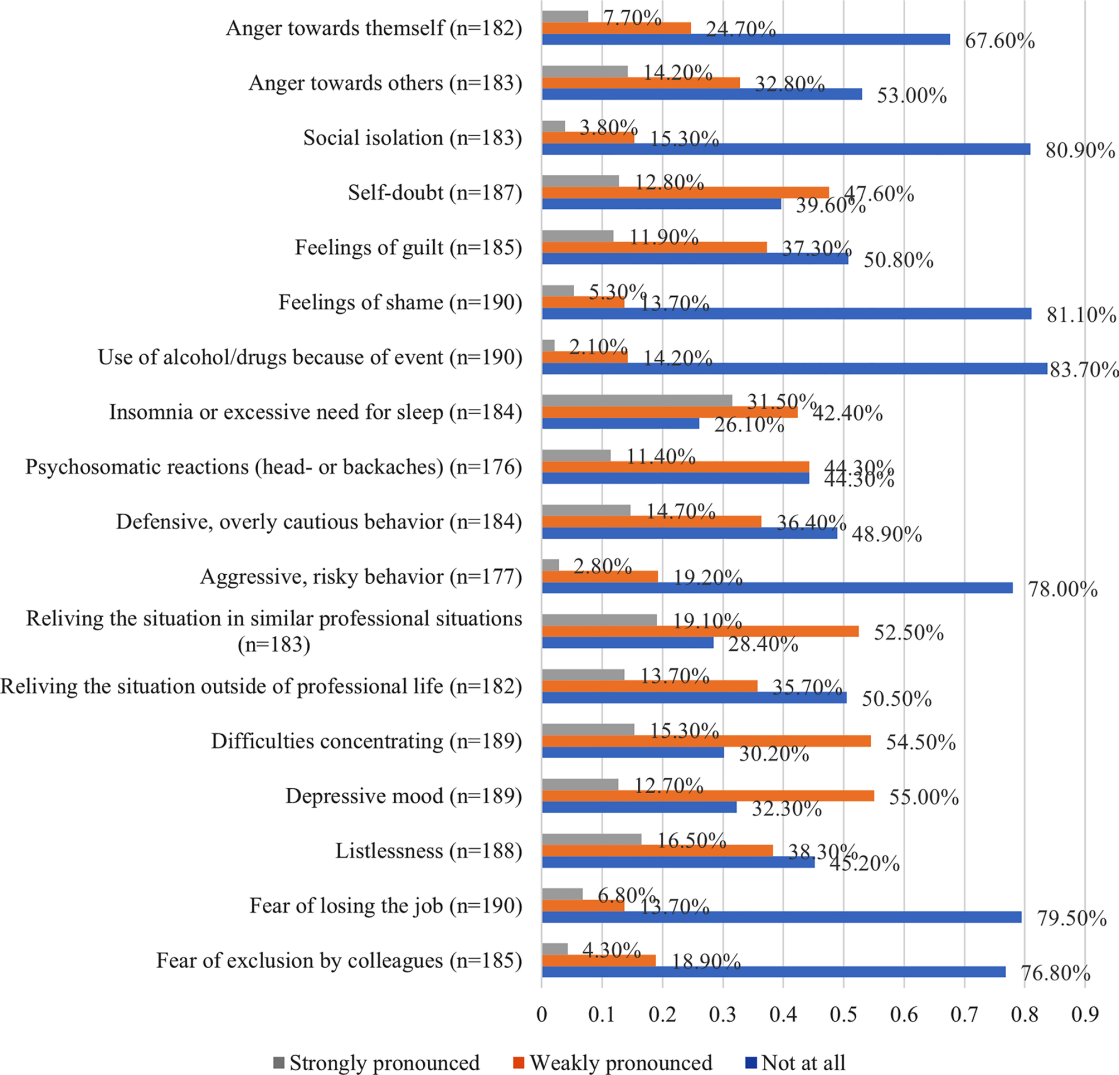
Symptoms of the self-identified second victims

Regarding recovery, most participants reported improvement within one week (55.6%, n = 109), while 21.4% recovered within one month (n = 42). Seven respondents (3.6%) indicated that recovery took one year or longer, and 21 participants (10.7%) reported not having fully recovered at the time of the survey. Figure 3 illustrates the distribution of reported symptoms among participants who identified themselves as Second Victims.”

Additionally, 17.8% (32/180) rated the desire for support by others to be strongly pronounced as well as the desire to debrief the incident for a better understanding to be strongly pronounced for 32.1% (59/184) of the participants. Most incidents were not related to COVID-19 (91.3%, *n* = 179/196)).

The ratings of support measures and differences between the ratings of Second Victims and Non-Second Victims are shown in Table 2 (1 = not helpful at all, 2 = not very helpful, 3 = rather helpful, 4 = very helpful).

**Table 2:** Ratings of support measures and differences between second victims (group 1) and non-second victims (group 2)

Support Measure	Not helpful at all % (*n*)	Rather not helpful % (*n*)	Rather helpful % (*n*)	Very helpful % (*n*)	*p*	*r*
**The possibility to take time off from work directly to process the event (*n*** **= 157)**	**2.50 (4)**	**15.30 (24)**	**12.70 (20)**	**69.40 (109)**	**< 0.01**	**-0.22**
Access to professional counseling or psychological/psychiatric consultations (crisis intervention) (*n* = 170)	1.20 (2)	11.20 (19)	8.80 (15)	78.80 (134)	0.78	-0.02
The possibility to discuss my emotional/ethical thoughts (*n* = 211)	2.40 (5)	21.80 (46)	8.50 (18)	67.30 (142)	0.86	-0.01
Clear and timely information regarding the course of action after a serious event (e.g., damage analysis, error report) (*n* = 184)	1.60 (3)	16.80 (31)	11.40 (21)	70.10 (129)	0.12	-0.12
Formal emotional support in the sense of organized collegial help (*n* = 212)	5.70 (12)	16.50 (35)	8.50 (18)	69.30 (147)	0.65	-0,03
Informal emotional support (*n* = 244)	3.70 (9)	22.10 (54)	13.50 (33)	60.70 (148)	0.74	-0.02
Quick processing of the situation/quick crisis intervention (in a team or individually) (*n* = 153)	0.70 (1)	13.10 (20)	11.10 (17)	75.20 (115)	0.07	-0.15
Support/Mentoring when continuing to work with patients (*n* = 256)	6.60 (17)	32.40 (83)	18.80 (48)	42.20 (108)	0.33	-0.06
Support when communicating with patients and/or relatives (*n* = 268)	7.50 (20)	32.10 (86)	14.90 (40)	45.50 (122)	0.79	-0.02
Guidelines regarding the role/activities expected of me after a serious event (*n* = 232)	5.20 (12)	21.10 (49)	11.20 (26)	62.50 (145)	0.19	-0.09
Support to be able to take an active role in the processing of the event (*n* = 223)	70.40 (157)	11.70 (26)	15.70 (35)	2.20 (5)	0.67	-0.03
A secure possibility to give information on how to prevent similar events in the future (*n* = 189)	4.20 (8)	12.20 (23)	10.10 (19)	73.50 (139)	0.24	-0.09
The possibility to access legal consultation after a severe event (*n* = 117)	76.90 (90)	13.70 (16)	6.80 (8)	2.60 (3)	0.16	-0.13

p: the p-value of the Mann-Whitney-U-test comparing the rating of the support measure between the group of participants who reported that they have already experienced SVP and the group of participants that reported that they have not experienced SVP in their career

A secure possibility to give information on how to prevent similar events in the future and access to professional counseling are rated highly as supportive measures, with no significant differences between Second Victims and Non-Second Victims. A significant difference between these two groups was found regarding the possibility to take time off from work directly to process the event: Second Victims significantly preferred this in comparison to Non-Second Victims (*p* < 0.01, *r*=-0.22). Due to this difference of ratings of support measures we accepted the corresponding hypothesis.

Our first binary logistic regression model investigated potential risk and protective factors for becoming a Second Victim. We found two associations: Professional experience has a positive association with Second Victim status, where each additional year of experience increases the odds by approximately 6% (OR = 1.06, 95% CI [1.03,1.10], *p* < 0.01). This result is statistically significant. The second significant factor associated with Second Victim status was the personality trait “openness”, where a higher score of 1 rank resulted in a 55.0% higher odds of becoming a Second Victim (OR = 1.55, 95% CI [1.18, 2.05], *p* < 0.01). The model demonstrated acceptable fit, with a Nagelkerke’s Pseudo-R² of 0.15 and an area under the curve (AUC) of 0.70, indicating moderate explanatory power and fair discriminative ability.

The only significant finding of our second binary logistic regression model investigating factors influencing overall symptom load was linked to neuroticism. Higher neuroticism values were linked with a significant increase of the odds to experience a high symptom load after experiencing SVP (OR = 1.57, 95% CI [1.07,2.31], *p* = 0.02). The overall model fit was modest, with a Nagelkerke’s Pseudo-R² of 0.13 and an area under the curve (AUC) below 0.6, indicating limited explanatory power and low discriminative ability of the model.

## Discussion

In this study, we explored the prevalence, symptom load, and preferred support measures for the SVP among EMDs. To our knowledge, this survey is the first to identify and quantify SVP specifically among EMDs, revealing a 62.2% self-reported prevalence. This work fills a critical gap in the literature, providing insights into the unique challenges faced by EMDs and the prevalence of SVP in this professional group. Our findings demonstrate that a significant proportion of EMDs self-identify as Second Victims, highlighting the emotional and psychological toll that their profession may impose.

The majority of respondents had no prior knowledge of SVP, yet over 60% identified themselves as Second Victims after learning about the phenomenon. This finding is consistent with previous studies that report high rates of SVP among healthcare professionals [12–14, 20]. For instance, studies among physicians described prevalence rates between 53% and 89% [12, 14, 20], while studies among nurses reported rates ranging from 60% to 81% [13, 21]. This comparison indicates that, despite not being physically present at the scene, EMDs are affected to a similar extent as frontline healthcare workers. This finding underscores the importance of recognizing EMDs as a professional group at risk of SVP and highlights the need for targeted support strategies comparable to those established in other healthcare settings. Supporting this, previous research has shown that EMDs are prone to experiencing stress, with exposure to emergency calls identified as a significant stressor [2, 6, 36–38]. In our study, the most commonly reported key events leading to SVP were unexpected deaths or suicides of patients, which aligns with literature showing that traumatic events with strong emotional impact are primary triggers for SVP [12–14, 20]. Moreover, Bedini et al. demonstrated that the severity of calls is associated with increased cortisol levels in EMDs, thereby heightening perceived stress [6]. Together, these findings suggest that the nature and intensity of emergency calls can contribute substantially to the development of SVP among EMDs.

Contrary to findings from previous SeViD-studies among physicians, nurses, and paramedics, colleagues did not represent the primary source of support for EMDs experiencing SVP [12–14, 19, 20]. Although peer support is widely recommended as an effective coping resource for Second Victims [9], most EMDs in our study reported turning to family and friends or to professional counseling services instead. This pattern suggests that the EMDs’ work environment, specifically operating alone in their individual workstations, may provide fewer opportunities for informal peer exchange or debriefing. Consequently, alternative organizational strategies are needed to strengthen support access for EMDs. The EMDs of this study also rated the access to professional counseling as highly helpful after experiencing traumatic events. Therefore, implementing structured employee assistance programs or low-threshold professional counseling services could help address this gap and improve post-incident recovery.

Our regression analysis identified two significant predictors of SVP, namely professional experience and the personality trait openness. Notably, those with more professional experience were more likely to become Second Victims. That result differs from the previous studies like SeViD-I, which found no such association [12]. Our finding that longer experience as an EMD constitutes a risk factor for SVP could be due to health care workers’ increased risk of experiencing at least one adverse event within their prolonged work life [39–41]. Additionally, higher openness was associated with an increased likelihood of experiencing SVP. It has previously been described that openness is positively associated with perspective-taking and empathy [42, 43]. This might explain a higher susceptibility to developing SVP within our sample. However, openness has not previously been linked to SVP, whereas other personality traits, such as neuroticism and extraversion, have been associated with a higher likelihood of experiencing SVP [13, 14, 20]. Therefore, the observed connection between SVP risk and openness may be coincidental and should be verified in future studies.

Neuroticism was identified as a significant risk factor for experiencing a higher symptom load following SVP, consistent with previous SeViD-studies [13, 20]. Neuroticism is characterized by a heightened tendency to experience negative emotions, thereby increasing an individual’s vulnerability to stress [28, 44]. Furthermore, neuroticism has been associated with lower resilience, reduced self-esteem, and less effective coping strategies, potentially limiting effective recovery from emotionally distressing events [30, 45]. These findings suggest that personality-related vulnerability factors, particularly neuroticism, may play an important role in shaping both the occurrence and severity of SVP symptoms among EMDs.

However, there are several limitations to our study. First, the cross-sectional design limits our ability to establish causal relationships between the identified factors and SVP [46]. The data reflect associations at a single point in time, which does not allow us to determine temporal order or directionality which is detrimental to establish causal relationships. Second, the use of a convenience sample may introduce selection bias, potentially overestimating the effect. Also, information on underlying psychiatric conditions was not collected. Consequently, we cannot exclude the possibility that pre-existing conditions influenced participants’ responses and were possible causes of reported symptoms. No correction for family-wise error rates has been carried out, as our approach was of purely exploratory nature. Despite this, the prevalence of SVP observed in our study is comparable to previous SeViD studies, suggesting that our findings are consistent with external evidence. Additionally, symptom load was rated by participants who identified themselves as Second Victims only. Previous findings from the SeViD-IX study indicated that even individuals who did not consider themselves Second Victims reported Second Victim-like symptoms, likely due to experiencing stressful events [47]. This suggests that there may be “hidden” or “silent” Second Victims in our sample, potentially leading to an underestimation of the total effect of SVP. Regarding our regression models it should be noted that the set of independent variables included in both models does not capture all potential confounders. Variables were chosen based on theoretical relevance and exploratory interest, but other unmeasured factors may also influence the occurrence of SVP. Further, follow-up questionnaires were not administered. This limits our ability to assess potential changes over time or the persistence of reported effects.

## Conclusion

The SeViD-VII study demonstrates that the Second Victim Phenomenon is highly prevalent among Emergency Medical Dispatchers in Germany, affecting more than half of the surveyed population. Despite their indirect involvement in patient care, EMDs experience emotional distress comparable to that of frontline healthcare professionals such as physicians, nurses, and paramedics. The findings highlight that professional experience and personality traits—particularly openness and neuroticism—play a significant role in the development and severity of SVP, suggesting that both cumulative exposure and individual vulnerability factors contribute to the phenomenon.

Unlike in previous SeViD studies, colleagues were not the primary source of support for affected EMDs; instead, they relied mainly on family, friends, or professional counseling. This underscores structural and social differences in the dispatcher work environment, where isolation and shift work may limit peer interaction and informal debriefing. Consequently, the establishment of structured support systems, such as employee assistance programs and accessible professional counseling, appears essential to facilitate recovery and resilience after distressing incidents.

Incorporating SVP awareness and coping strategies into dispatcher training could enhance preparedness and reduce the long-term impact of emotionally demanding calls. Future research should develop and test targeted intervention models, and further investigate personality-related risk and resilience factors. By addressing these aspects, healthcare systems can strengthen psychological support and occupational health for this often-overlooked yet crucial professional group.

## Supplementary Information

Below is the link to the electronic supplementary material.

Supplementary Material 1

Supplementary Material 2

## Data Availability

The datasets used and/or analyzed during the current study are available from the corresponding author on reasonable request.

## Footnotes

1
https://www.fvlst.de/.

2
https://www.dgre.org/.

## Abbreviations

BFI: Big Five Inventory
CI: Confidence Interval
EMD: Emergency medical dispatcher
M: Mean
OR: Odds ratio
SD: Standard deviation
SeViD: Second Victims in German-speaking Countries
SVP: Second Victim Phenomenon
