# Supplementary material for: Second victims among emergency medical dispatchers in Germany: a cross-sectional study (SeViD-VII)
Source: Int J Emerg Med. 2025 Dec 22;19:27. doi: 10.1186/s12245-025-01084-y (PMC12838509; doi:10.1186/s12245-025-01084-y)
Supplement: Supplementary file 1 — Supplementary Material 1 [file 12245_2025_1084_MOESM1_ESM.pdf]

Questionnaire on „Second Victims“

Part 1: Experience with critical situations

1.1 Prior to our survey, were you aware of the term “Second Victim”, which describes a person in health care who has been emotionally traumatized by an unexpected clinical event?

- ◻ Yes
- ◻ No

1.2 Have you ever experienced this sort of traumatization after an event in the course of your professional work in health care?

- ◻ Yes, once → Continue with question 1.3
- ◻ Yes, more than once → Continue with question 1.3
- ◻ No → Continue with question 1.9

1.3 Did this event (if multiple, at least one of them) occur <u>within the last 12 months</u>?

- ◻ Yes
- ◻ No

1.4 If you experienced multiple critical events, please think of the most formative of these events (key event). Regardless whether it was caused by you. What type of event is it?

- ◻ Incident with patient harm
- ◻ Incident without patient harm/near miss
- ◻ Unexpected death/suicide of a patient
- ◻ Unexpected death/suicide of a colleague
- ◻ Aggressive behavior of a patient/relative
- ◻ Other (please explain): ______________________

1.5 Did you receive support from others after this key event?

- ◻ Yes
- ◻ No, even though I asked for help
- ◻ No, I haven’t asked anyone for help

1.6 If you received support from others, please indicate by which group(s).

- ◻ Colleagues
- ◻ Superiors
- ◻ Management
- ◻ Family/Friends
- ◻ Pastoral care/Psychological counseling/Psychotherapy

1.7 How long did it take you to fully recover from this key event?

- ◻ Less than a day
- ◻ Within a week
- ◻ Within a month
- ◻ Within a year
- ◻ More than a year
- ◻ I have not fully recovered, yet

1.8 Every person who has experienced or witnessed a traumatic event, experiences and processes it differently. However, the following reactions are quite typical according to research findings. How pronounced were the following reactions to the traumatic event for you?

	Strongly pronounced	Weakly pronounced	Not at all	I don't know
Fear of exclusion by colleagues	☐	☐	☐	☐
Fear of losing the job	☐	☐	☐	☐
Listlessness	☐	☐	☐	☐
Depressive mood	☐	☐	☐	☐
Concentration difficulties	☐	☐	☐	☐
Reliving the situation outside the professional activity	☐	☐	☐	☐
Reliving the situation in similar professional situations	☐	☐	☐	☐
Aggressive, intentionally risky behavior	☐	☐	☐	☐
Defensive, overly cautious behavior	☐	☐	☐	☐
Psychosomatic reactions (headaches, back pain, etc.)	☐	☐	☐	☐
Insomnia or excessive need for sleep	☐	☐	☐	☐
Use of alcohol/drugs due to the event	☐	☐	☐	☐
Feelings of shame	☐	☐	☐	☐
Feelings of guilt	☐	☐	☐	☐
Self-doubt	☐	☐	☐	☐
Social isolation	☐	☐	☐	☐
Anger/rage towards others	☐	☐	☐	☐
Anger/rage towards myself	☐	☐	☐	☐
Desire for support by others	☐	☐	☐	☐
Desire to process the event for better understanding	☐	☐	☐	☐

1.9 Whether or not you yourself have experienced a traumatizing event, we ask you to rate how helpful you find the support measures suggested in the literature for yourself or your colleagues

	Very helpful	Rather helpful	Rather not helpful	Not helpful at all	I cannot judge this
The possibility to take time off from work directly to process the event	☐	☐	☐	☐	☐
Access to professional counseling or psychological/psychiatric consultation	☐	☐	☐	☐	☐
The possibility to discuss my emotional or ethical thoughts	☐	☐	☐	☐	☐
Clear and timely information regarding the course of action after a serious event (e.g., damage analysis, error report)	☐	☐	☐	☐	☐
Formal emotional support in the sense of organized collegial help	☐	☐	☐	☐	☐
Informal emotional support	☐	☐	☐	☐	☐
Quick processing of the situation/quick crisis intervention (in a team or individually)	☐	☐	☐	☐	☐
Support/Mentoring when continuing to work with patients	☐	☐	☐	☐	☐
Support when communicating with patients and/or relatives	☐	☐	☐	☐	☐
Guidelines regarding the role/activities expected of me during a serious event	☐	☐	☐	☐	☐
Support to be able to take an active role in the processing of the event	☐	☐	☐	☐	☐
A secure possibility to give information on how to prevent similar events in the future	☐	☐	☐	☐	☐
The possibility to access legal consultation after a severe event	☐	☐	☐	☐	☐

Part 2: Last page of the questionnaire

Do you have any comments or remarks about this questionnaire? We are happy about every hint!

Selection options: optional free text field

You have completed the survey! Thank you for your participation!

Strametz, R., Rösner, H., Ablöscher, M. *et al.* Entwicklung und Validation eines Fragebogens zur Beurteilung der Inzidenz und Reaktionen von Second Victims im Deutschsprachigen Raum (SeViD). *Zbl Arbeitsmed* **71**, 19–23 (2021). https://doi.org/10.1007/s40664-020-00400-y
