# Supplementary material for: Second victims among emergency medical dispatchers in Germany: a cross-sectional study (SeViD-VII)
Source: Int J Emerg Med. 2025 Dec 22;19:27. doi: 10.1186/s12245-025-01084-y (PMC12838509; doi:10.1186/s12245-025-01084-y)
Supplement: Supplementary file 2 — Supplementary Material 2 [file 12245_2025_1084_MOESM2_ESM.pdf]

BFI-10 by Rammstedt et al.

Item	Polarity	Subscales
I see myself as someone who is reserved.	-	Extraversion
I see myself as someone who is generally trusting.	+	Agreeableness
I see myself as someone who tends to be lazy	-	Conscientiousness
I see myself as someone who is relaxed, handles stress well.	-	Neuroticism
I see myself as someone who has few artistic interests.	-	Openness
I see myself as someone who is outgoing, sociable.	+	Extraversion
I see myself as someone who tends to find fault with others.	-	Agreeableness
I see myself as someone who does a thorough job.	+	Conscientiousness
I see myself as someone who gets nervous easily.	+	Neuroticism
I see myself as someone who has an active imagination.	+	Openness

Respondents can choose from a five-point rating scale ranging from “does not apply at all” (1) to “applies completely” (5).

Rammstedt, B., Kemper, C. J., Klein, M. C., Beierlein, C., & Kovaleva, A.(2014). Big Five Inventory (BFI-10). *Zusammenstellung sozialwissenschaftlicher Items und Skalen (ZIS)*. https://doi.org/10.6102/zis76
